# Echocardiographic predictors of first onset of atrial fibrillation in dogs with myxomatous mitral valve disease

**DOI:** 10.1111/jvim.15860

**Published:** 2020-08-07

**Authors:** Marco Baron Toaldo, Chiara Mazzoldi, Giovanni Romito, Helen Poser, Barbara Contiero, Mario Cipone, Carlo Guglielmini

**Affiliations:** ^1^ Department of Veterinary Medical Sciences Alma Mater Studiorum ‐ University of Bologna Bologna Italy; ^2^ Department of Animal Medicine, Production and Health University of Padova Padua Italy; ^3^ Present address: Division of Cardiology, Clinic for Small Animal Internal Medicine Vetsuisse Faculty University of Zürich Zürich Switzerland

**Keywords:** canine, left atrium, speckle tracking echocardiography, strain

## Abstract

**Background:**

Atrial fibrillation (AF) occurs in dogs with myxomatous mitral valve disease (MMVD) as a consequence of left atrial (LA) dilatation, and it affects survival and quality of life.

**Objectives:**

To evaluate the usefulness of echocardiography in predicting the first occurrence of AF in dogs with MMVD.

**Animals:**

Forty‐four client‐owned dogs with MMVD, 22 dogs that developed AF, and 22 dogs that maintained sinus rhythm.

**Methods:**

Retrospective observational study. Medical databases were reviewed for dogs that developed AF during the year after diagnosis of MMVD (AF group). The last echocardiographic examination obtained while still in sinus rhythm was used to derive selected variables. For each dog with AF, a control dog matched for body weight, class of heart failure, and LA dimension was selected. Echocardiographic results including LA volumes and LA speckle tracking echocardiography (STE)‐derived variables were measured.

**Results:**

Among the tested echocardiographic variables, only LA diameter (*P* = .03) and left ventricular internal diameter in diastole (*P* = .03) differed significantly between groups, whereas body weight‐indexed variables of cardiac dimension as well as LA volumes and volume‐derived functional variables were not different. Among the STE‐derived variables, peak atrial longitudinal strain (PALS) results differed significantly between the AF group (23.8% ± 8.6%) and the control group (30.5% ± 9.6%; *P* = .03). A value of PALS ≤28% predicted AF occurrence with sensitivity and specificity of 0.80 and 0.65, respectively.

**Conclusions and Clinical Importance:**

Absolute cardiac diameters and LA STE (in particular, PALS) are useful echocardiographic predictors for the development of AF in dogs with MMVD.

**Abbreviations**
AF: atrial fibrillation
BW: body weight
CHF: congestive heart failure
HR: heart rate
LA: left atrial
LAP: left atrial volume at the onset of the P wave
LAV max: maximal left atrial volume
LAV min: minimal left atrial volume
LV: left ventricular
MMVD: myxomatous mitral valve disease
NSR: normal sinus rhythm
PACS: peak atrial contraction strain
PALS: peak atrial longitudinal strain
ROC: receiver operating characteristic
STE: speckle tracking echocardiography

## 1 INTRODUCTION

Atrial fibrillation (AF) is a common arrhythmia in dogs with underlying cardiac disease associated chamber dilatation.^1^ Dogs without underlying cardiac disease have a better prognosis than those with cardiac disease, regardless of whether or not it is associated with congestive heart failure (CHF).^1^ Because dogs with left atrial (LA) dilatation and higher body weight (BW) are at increased risk of developing AF, this arrhythmia is detected most frequently in large breed dogs with dilated cardiomyopathy.^1^, ^2^, ^3^ However, when present in medium‐to‐large‐breed dogs with myxomatous mitral valve disease (MMVD), AF represents an independent risk factor for cardiac death.^4^ Ventricular response rate is another pivotal factor affecting clinical presentation and outcome in dogs with AF. Animals with increased heart rate (HR), as measured instantaneously or by calculating the mean HR over 24 hours using a Holter monitor, or poor pharmacological rate control have worse outcome.^1^, ^4^, ^5^ Therefore, appropriate control of mean HR using nodal blockers, such as digoxin and diltiazem, should be carried out to increase life expectancy of these patients.^5^ Thus, early detection of AF in dogs with cardiac disease is extremely important in order to begin effective treatment and improve survival.

In people, several studies have evaluated the usefulness of surface ECG and transthoracic echocardiography in predicting the future occurrence of AF.^6^, ^7^, ^8^, ^9^ Among these, advanced echocardiographic techniques such as LA tissue Doppler imaging and LA speckle tracking echocardiography (STE) have been shown to be valuable tools for predicting AF occurrence in humans with cardiac disease or normally‐structured hearts.^10^, ^11^, ^12^, ^13^ Left atrial tissue Doppler imaging and STE also have been applied in both clinically healthy dogs and dogs with MMVD.^14^, ^15^, ^16^, ^17^, ^18^, ^19^ In particular, LA STE‐derived strain has been shown to decrease progressively as the disease advances,^15^ and it is a predictor of cardiac death in dogs with MMVD.^17^

Our aim was to evaluate the clinical usefulness of several indices of LA and left ventricular (LV) morphology and function to predict the future occurrence of AF in a population of dogs with naturally occurring MMVD.

## 2 MATERIALS AND METHODS

### 2.1 Animals

The medical databases of the veterinary teaching hospitals of the Universities of Bologna and Padova were retrospectively reviewed from January 2011 to December 2018 searching for all dogs with MMVD that developed AF during the 12 months after diagnosis of MMVD and inclusion in the study (AF group). Only dogs with at least 1 echocardiographic examination performed before the occurrence of AF that showed normal sinus rhythm (NSR) were considered. The diagnosis of AF was made by analyzing a 6‐ or 12‐lead surface ECG by a board‐certified cardiologist. Information regarding clinical variables including breed, sex, BW, and age; presence or absence of CHF; class of heart failure^20^; drugs received; and the time between the last echocardiographic examination performed in NRS and the diagnosis of AF were recorded. Echocardiographic examinations were retrieved from a dedicated server as digital imaging and communications in medicine (DICOM) files and analyzed by a single operator using dedicated software for image analysis (OsiriX MD Software, Pixeo SARL, Geneva, Switzerland; QLAB quantification software version 9.1, Philips Healthcare, Monza, Italy). A control group was created by selecting dogs with MMVD during the same period of time. These animals matched those of the AF group based on similar signalment, same heart failure class, and similar LA dimension as expressed as the ratio between the diameter of the left atrium and aorta described below. Dogs in the control group had a follow‐up time that was longer than the time between the last echocardiographic examination in NSR and the occurrence of AF for the corresponding patient in the AF group.

### 2.2 Echocardiography

Transthoracic echocardiographic examinations were reviewed and analyzed by a board‐certified cardiologist to confirm the diagnosis of MMVD and obtain LA and LV morphological and functional variables as well as Doppler‐derived variables. All examinations were performed using echocardiographic units (iE33 and CX50 ultrasound systems, Philips Healthcare) from the same manufacturer with similar software and technology, and equipped with a dedicated multifrequency phased array transducer (S5‐1 pure‐wave phased array transducer, Philips Healthcare). Echocardiographic diagnosis of MMVD was made by observing typical thickening, prolapse of the mitral valve, or insufficiency of the mitral valve on color Doppler examination, or a combination of these. Wall thickness and chamber diameter of the LV were obtained from 2‐dimensional guided M‐mode images acquired from a right parasternal short axis view at the level of the papillary muscles. In particular, LV end‐diastolic and end‐systolic dimensions were normalized to BW and compared to previously published reference intervals.^21^ Fractional shortening then was calculated using the appropriate formula. The LA diameter and aortic diameter were measured on 2‐dimensional images obtained from a right parasternal short axis view at the basilar level, as previously described.^22^ The LV diastolic inflow was recorded from the left apical 4 chamber view by placing the sample volume at the level of the mitral valve on the ventricular side. The peak velocity of the early diastolic flow wave, peak velocity and duration of the late diastolic flow wave, and ratio between the 2 peak velocities were measured.

The LA volumes were measured only from a left apical 4‐chamber view using the area‐length method, as previously described.^23^ The following variables were calculated: maximal LA volume (LAV max); minimal LA volume (LAV min); LA volume at the onset of the P wave (LAP); LA total emptying volume (LAV max − LAV min); LA expansion index ([LAV max − LAV min]/LAV min); LA passive emptying volume (LAV max − LAP); LA passive emptying percentage of total emptying ([LAV max − LAP]/[LAV max − LAV min]); LA active emptying volume (LAP − LAV min); LA active emptying percentage of total emptying ([LAP − LAV min]/[LAV max − LAV min]). Finally, LAV max, LAV min, and LAP indexed to BW were calculated by dividing these variables by the BW of the dog expressed in kilograms.

The LA STE was performed as described in a previous study.^15^ Briefly, from a left apical 4‐chamber view, 3 points were selected on the left atrium (2 at the mitral valve annulus and 1 on the atrial roof) and the software automatically drew a region of interest with 7 different subregions. The perfect track of the segments of the LA walls was manually evaluated and edited as needed. The time/strain curves then were generated, and the following values were annotated: peak atrial longitudinal strain (PALS; representing the peak strain value during LV contraction); peak atrial contraction strain (PACS; representing the strain value just before LA active contraction on the peak of the P wave of the ECG trace); and contraction strain index, calculated using the formula: ([PACS/PALS] × 100).

All measurements were replicated on 3 consecutive beats, and the mean values then were calculated. The HR provided by the continuous ECG recording performed during each echocardiographic examination was annotated.

### 2.3 Variability study

Interobserver and intraobserver variability of LA STE already has been studied in dogs with MMVD, and it appeared to be clinically acceptable.^15^ Because 2 different echocardiographic units were used at 2 enrollment centers, the effect of the echocardiographic machine was evaluated by selecting 5 dogs not included in the study population. In particular, 5 dogs with or without MMVD were recruited from the university students and hospital staff, and a complete echocardiographic examination was repeated using both machines during the same day in the same dogs, approximately 1 hour apart. At the end of the variability study, the operator performed the STE analysis on the 10 studies that were anonymized to keep the operator blind to the dogs' identities. Variability then was calculated using the coefficient of variation, as previously described.^24^

### 2.4 Statistical analysis

All statistical analyses were performed using 2 commercial dedicated software programs (Prism 7, GraphPad Software Inc, San Diego, California; SAS version 9.3, SAS Institute Inc, Cary, North Carolina). Distribution of continuous data was assessed visually and by using a D'Agostino‐Pearson normality test. Normally distributed data are expressed as mean ± SD, whereas nonnormally distributed variables are presented as median (interquartile range [IQR]). Continuous variables were compared between groups using an unpaired *t* test or a Mann‐Whitney test as needed, whereas categorical data were compared using a Chi‐squared test. A univariate analysis was performed to determine whether a significant relationship existed between echocardiographic variables and future occurrence of AF. Predictors with *P* < .1 were retained for the subsequent multivariable analysis. In this second step, the variables were considered to significantly affect outcome when *P* < .05. The relative risk and the 95% confidence interval (CI) were calculated. Receiver operating characteristic (ROC) curves were obtained by plotting sensitivity versus specificity to determine the ability of each echocardiographic variable to predict future AF occurrence. The area under the curve of the ROC curves for each variable was compared. Moreover, for each variable, the optimal cutoff corresponding to the value closest to the upper left corner of the graph was identified (Youden criterion). Sensitivity and specificity were calculated by use of the cutoffs determined from the ROC curves. A value of *P* < .05 was considered significant for all analyses.

## 3 RESULTS

### 3.1 Demographic variables

Forty‐four dogs were included in the study (22 dogs for each group). No difference was found between groups for BW (*P* = .8), age (*P = .99*), and sex (*P* = .5; Table 1). In the AF group, the most common breed was the mixed breed (14/22 dogs, 63.6%), followed by 1 each of the following breeds: Dachshund, Cavalier King Charles spaniel, Springer Spaniel, Cocker Spaniel, Jack Russell Terrier, Drahthaar, Italian Griffon, and Border Collie. In the control group, the most common breed was the mixed breed (16/22 dogs, 72.7%), followed by Dachshund and Beagle (2/22 dogs each, 9.1%), and 1 each of the Shar‐Pei and Bolognese (4.5%). No difference in breed distribution was found between groups (*P* = .75). The classes of heart failure were perfectly matched between the 2 groups, with 17 dogs (77.3%) in each group being in the symptomatic class (stage C), 3 dogs (13.6%) in a compensated state with cardiomegaly (stage B2), and 2 dogs (9.1%) in a compensated state without cardiomegaly (stage B1). Drugs used for the treatment of the underlying valvular disease were furosemide, pimobendan, benazepril, spironolactone, enalapril, hydrochlorothiazide, torasemide, amlodipine, sotalol, and mexiletine. No difference in terms of drugs administered between the 2 groups was found (*P* = .9). Median time from the last echocardiographic examination and the occurrence of AF in the study group (142.5 days; IQR, 64‐324.3 days) was significantly shorter compared to the follow‐up time of dogs in the control group (median, 291 days; IQR, 178.3‐566.3 days; *P* = .01).

**TABLE 1:** Signalment and echocardiographic variables in a population of 44 dogs with myxomatous mitral valve disease that developed or not atrial fibrillation during the following year

Variable	Control group (n = 22)	AF group (n = 22)	*P* value
BW (kg)	13.0 (8.4‐18.0)	11.4 (9.2‐20.4)	.80
Age (years)	11.5 ± 2.1	11.5 ± 2.0	.99
Last echo ‐ event (days)	291.0 (178.3‐566.3)	142.5 (64.0‐324.3)	.01
Heart rate (bpm)	137.6 ± 28.4	149.6 ± 32.9	.20
LA/Ao	2.2 ± 0.5	2.5 ± 0.5	.09
LA diam (mm)	38.1 (35.8‐43.7)	42.3 (38.9‐50.2)	.03
Ao (mm)	18.6 ± 4.2	18.6 ± 4.2	.99
E wave (m/s)	1.2 (1.0‐1.5)	1.5 (1.1‐1.6)	.12
A wave (m/s)	0.7 ± 0.2	0.7 ± 0.2	.95
E/A	2.0 (1.4‐2.4)	2.0 (1.7‐3.0)	.34
A wave dur (ms)	82.3 ± 12.8	80.4 ± 17.0	.69
LVIDD (mm)	42.0 (40.2‐48.1)	47.2 (43.95‐52.05)	.03
LVIDS (mm)	24.6 (21.93‐28.5)	26.5 (22.08‐29.08)	.50
LVIDDn	2.1 ± 0.4	2.3 ± 0.4	.13
LVIDSn	1.1 (1.0‐1.2)	1.1 (1.0‐1.3)	.41
LV FS (%)	43.3 ± 6.9	44.1 ± 8.1	.72
LAVmax (mL)	62.8 ± 37.4	74.0 ± 49.1	.40
LAVmin (mL)	32.4 ± 25.7	39.5 ± 31.9	.42
LAVp (mL)	42.8 ± 26.2	54.7 ± 46.6	.30
LAVmax:BW (mL/kg)	4.8 ± 2.7	5.5 ± 3.2	.49
LAVmin:BW (mL/kg)	2.5 ± 1.8	2.9 ± 2.2	.52
LAVp:BW (mL/kg)	3.3 ± 1.9	3.9 ± 2.7	.37
LAEV (mL)	30.5 ± 14.7	34.5 ± 25.2	.52
LAEi (%)	120.1 ± 57.6	121.1 ± 85.6	.96
LAPEV (mL)	20.0 ± 12.8	19.3 ± 13.0	.85
LAPE (%)	63.4 ± 15.5	59.4 ± 20.6	.46
LAAEV (mL)	10.5 ± 5.8	15.2 ± 20.8	.31
LAAE (%)	36.6 ± 14.5	40.7 ± 20.6	.46
Strain PALS (%)	30.5 ± 9.6	23.8 ± 8.6	.03
Strain PACS (%)	7.0 (2.0‐12.8)	4.5 (2.3‐8.5)	.31
CSI (%)	24.4 ± 16.8	22.9 ± 13.2	.76

*Note:* Normally and not normally distributed data are presented as mean ± SD and median (interquartile range), respectively.

Abbreviations: A wave, mitral valve A wave peak velocity; A wave dur, A wave duration; AF, atrial fibrillation; Ao, aorta diameter; BW, body weight; CSI, contraction strain index; E wave, mitral valve E wave peak velocity; E/A, mitral valve peak E and A wave velocity ratio; LA diam, left atrial diameter; LA/Ao, left atrial diameter to aorta ratio; LAAE, left atrial active emptying percentage of total emptying; LAAEV, left atrial active emptying volume; LAEi, left atrial expansion index; LAEV, left atrial emptying volume; LAPE, left atrial passive emptying percentage of total emptying; LAPEV, left atrial passive emptying volume; Last echo‐event, time passed from the last echocardiographic exam to development of atrial fibrillation/last contact; LAVmax, left atrial maximal volume; LAVmin, left atrial minimal volume; LAVp, left atrial volume on P wave; LV FS, left ventricular fractional shortening; LVIDD, left ventricular internal diameter in diastole; LVIDDn, left ventricular internal diameter in diastole indexed to body weight; LVIDS, left ventricular internal diameter in systole; LVIDSn, left ventricular internal diameter in systole indexed to body weight; strain PACS, peak atrial contraction strain; strain PALS, peak atrial longitudinal strain.

### 3.2 Echocardiography

Among the conventional echocardiographic variables, LA diameter was higher in dogs of the AF group (median, 42.3 mm; IQR, 38.9‐50.2 mm) compared to that of dogs of the control group (median, 38.5 mm; IQR, 35.8‐43.7 mm; *P* = .03). Similarly, LV internal diameter at end diastole was larger in dogs of the AF group (median, 47.2 mm; IQR, 44.0‐52.1 mm) compared to that of dogs of the control group (median, 42.0 mm; IQR, 40.2‐48.1 mm; *P* = .03). None of the Doppler‐derived variables, LA volumes, and LA volume‐derived functional variables differed between the 2 groups. Among the STE‐derived variables, mean PALS was lower in the AF group (23.8% ± 8.6%) compared to that of dogs of the control group (30.5% ± 9.6%; *P* = .03; Table 1).

**FIGURE 1:**
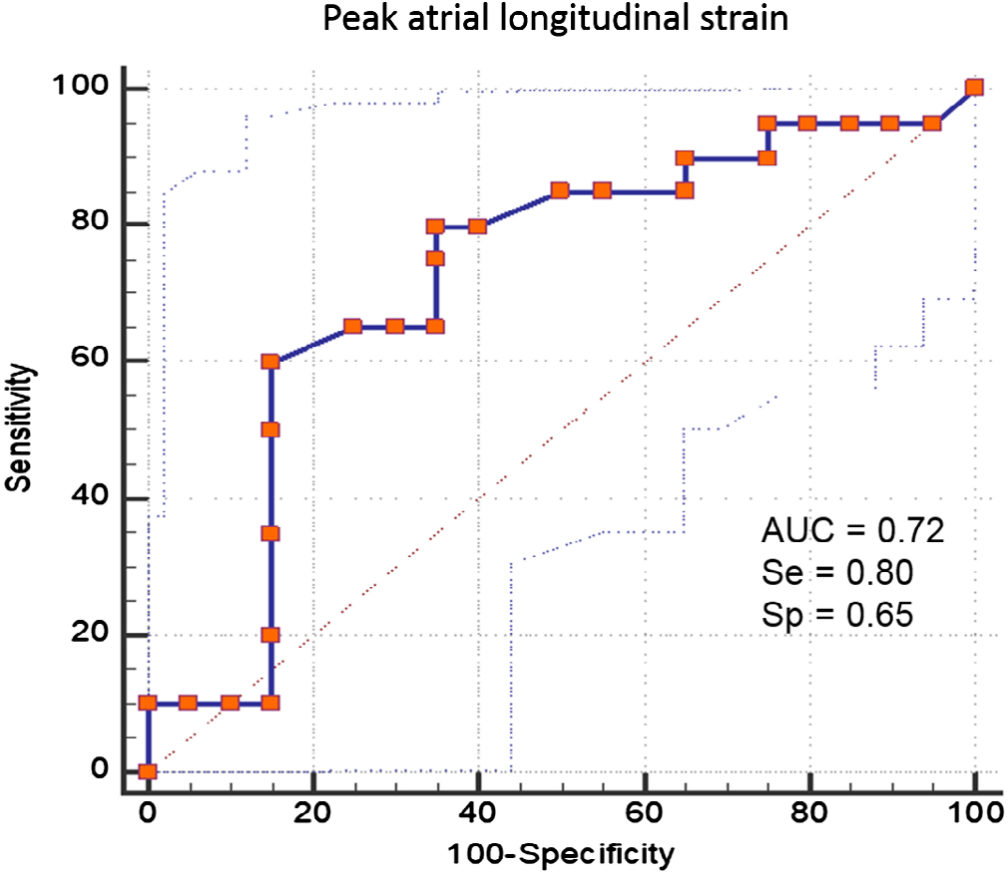
Receiver operating characteristic curve for peak atrial longitudinal strain for the prediction of first occurrence of atrial fibrillation in a population of 44 dogs with myxomatous mitral valve disease. The area under the curve represented by the thicker line is 0.721 (95% confidence interval, 0.555‐0.888). The overall most accurate cutoff point identified on the curve was peak atrial longitudinal strain ≤28%, which had a sensitivity and a specificity of 0.80 and 0.65, respectively

Univariate analysis of the effect of echocardiographic variables identified LA/Ao (*P* = .06), LA diam (*P* = .09), and PALS (*P* = .03) as potential predictors of AF. Because of multicollinearity among these predictors, the multivariable analysis did not converge. The only variable having a significant effect on the future development of AF was PALS, with a Wald chi‐square of 4.76 and relative risk of 0.96 (95% CI, 0.93‐0.99). Because PALS was the only variable showing significant impact on the development of AF, ROC curve was created for this variable only. The area under the ROC curve was 0.721 (SE, 0.085; 95% CI, 0.555‐0.888), and the optimal cutoff to predict future onset of AF was ≤28%, with sensitivity and specificity of 0.80 and 0.65, respectively (Figure 1; Table 2).

**TABLE 2:** Diagnostic accuracy of peak atrial longitudinal strain in predicting first occurrence of atrial fibrillation in dogs with myxomatous mitral valve disease

AUC ± SE	95% confidence interval	Cutoff	Sensitivity	Specificity
0.721 ± 0.085	0.555‐0.888	≤47%	0.95	0.05
		≤28%	0.80	0.65
		≤12%	0.10	1.00

Abbreviation: AUC, area under the curve.

### 3.3 Variability study

The variability between the 2 echocardiographic machines was clinically acceptable (<15%) for all STE‐derived variables. In particular, the corresponding coefficients of variation were 12.9, 14.8, and 11.0% for PALS, PACS, and the contraction strain index, respectively.

## 4 DISCUSSION

We showed that, in dogs with MMVD, absolute LA and LV dimensions are higher in those subjects that develop AF during the year after the echocardiographic examination. However, only STE‐derived PALS represents an independent predictor of AF development in these dogs after univariate analysis, with an increased risk of arrhythmia when PALS ≤28%.

The occurrence of AF in dogs with CHF usually is the result of LA remodeling with chamber dilatation and interstitial fibrosis.^25^ Because a critical atrial mass is needed to perpetuate the arrhythmia,^26^ dilatation of the left atrium usually is present in small animals. This factor is even more important in small breed dogs, such as those that typically are affected by MMVD, in which LA dilatation usually accompanies the most advanced stages of the disease. Body weight is a risk factor for the development of AF in dogs,^2^ and giant breeds usually are more frequently affected by the arrhythmia.^1^ In our study, all dogs with MMVD were included, which is a different approach from that of a previous study that evaluated the prognostic relevance of AF in medium‐to‐large‐breed dogs (>15 kg) with MMVD.^4^ Therefore, the animals included in our study had severely dilated LA likely associated with relevant atrial remodeling and fibrosis that allowed for maintenance of the arrhythmia. Therefore, studying LA dimension using echocardiography can give relevant information about the stratification of patients with cardiac disease, because several studies in people have indicated that LA enlargement represents a risk factor for development of cardiac events, including first onset of AF.^8^, ^27^, ^28^, ^29^

The dilatation of the LA is not a simple elongation of the myocardial fibers, but an active process of myocardial remodeling. This remodeling implies myofiber hypertrophy, changes in the interstitial matrix, and fibrosis.^30^, ^31^, ^32^ In a canine model of CHF induced by tachy‐pacing, the heart failure itself promoted atrial interstitial fibrosis, with subsequent abnormal local conduction and occurrence of sustained AF.^25^ This process, together with abnormal LA afterload and diastolic dysfunction of the left ventricle, leads not only to enlargement of the chamber, but also to abnormal systolic properties.^33^, ^34^ In people, abnormal LA function represents a stronger predictor of AF occurrence when compared to echocardiographic evaluation of LA dimensions alone.^35^ Thus, several echocardiographic variables reflecting LA systolic function have been used to predict the occurrence of AF. In particular, volume‐derived functional variables, such as total, active, and passive ejection fractions can be useful for detecting LA dysfunction.^34^, ^35^ Moreover, Doppler‐derived transmitral wave flow velocities and velocity time integrals can be used not only to assess LV diastolic function, but also to estimate LA booster function.^35^ In our study of dogs with MMVD, none of these variables differed between animals that developed AF and those that maintained NSR. Moreover, in other studies performed in dogs with MMVD, none of the LA volume‐ and area‐derived functional variables predicted cardiac‐related death and occurrence of CHF.^17^, ^36^ Therefore, these echocardiographic variables seem to have limited value in detecting clinically relevant LA dysfunction in dogs with MMVD.

Left atrial strain can be analyzed using the tissue Doppler technique or STE software. Both methods have been used in dogs, and the latter shows better reproducibility and clinical relevance.^14^, ^15^ In a recent study performed on dogs with different types of cardiac disease, tissue Doppler appeared to be useful in predicting the future onset of AF.^37^ In particular, the time between the onset of the P wave on the ECG and the peak of the last diastolic wave recorded on the mitral annulus differed between dogs that developed AF in the following months and those that did not.^37^ This variable is an indicator of atrial electromechanical delay, but does not directly reflect the systolic properties of the chamber.^34^ Left atrial tissue Doppler imaging, however, is an angle‐dependent technique that suffers from several limitations and has limited clinical use in veterinary medicine.^14^ In contrast, the STE‐derived deformation technique is more reproducible and clinically useful. In dogs with MMVD, PALS progressively decreases while heart failure class advances and LA dilatation becomes worse.^16^, ^18^ Moreover, lower PALS values indicate increased risk of cardiac death in dogs with MMVD.^17^ Therefore, PALS is a valuable echocardiographic variable to noninvasively assess LA mechanical dysfunction in dogs with cardiac disease.

In people with AF, peak reservoir strain and strain rate inversely predict the extent of LA fibrosis, as assessed by cardiac magnetic resonance imaging.^12^ Therefore, because interstitial fibrosis plays an important role in the occurrence and maintenance of AF, STE variables can be helpful in better characterizing the disease process and pathologic condition of the LA. Moreover, several studies in people now have shown that STE variables can identify subtle dysfunction of the LA and predict future occurrence (or reoccurrence) of AF independently of other echocardiographic variables.^10^, ^11^, ^12^, ^13^, ^38^ In our study, a decrease in PALS was the only variable that could predict future occurrence of AF in the univariate model. Because all of the other echocardiographic variables reflecting LA volume did not differ between groups, PALS might indicate the presence of more severe myocardial fibrosis in those dogs that developed arrhythmia in the subsequent months. Although no therapeutic strategies have been clinically useful to prevent occurrence of AF in dogs, recognition of increased risk for arrhythmia development can suggest the need for more frequent evaluation and treatment optimization for concomitant cardiac diseases.

Our study has some limitations. First, the number of dogs enrolled was low, which was a consequence of the difficulty of locating patients with MMVD that developed AF during the follow‐up time and the low prevalence of arrhythmia in this specific population of animals. Second, although LA STE has been determined to be feasible and reproducible in dogs,^15^, ^18^ the technique requires high‐quality images, dedicated software, and expertise for the analysis. Moreover, LV STE has been shown to produce different results according to the manufacturer of the machine used for the analysis.^39^ Thus far, it is unknown if this phenomenon also is true for LA STE, but it is possible that studies performed using different software will deliver different results.

In conclusion, we showed that, together with absolute LA and LV diameters, the use of LA STE could predict the future development of AF in dogs with MMVD. In particular, PALS was the best predictor of AF in these animals.

## CONFLICT OF INTEREST DECLARATION

Authors declare no conflict of interest.

## OFF‐LABEL ANTIMICROBIAL DECLARATION

Authors declare no off‐label use of antimicrobials.

## INSTITUTIONAL ANIMAL CARE AND USE COMMITTEE (IACUC) OR OTHER APPROVAL DECLARATION

Authors declare no IACUC or other approval was needed.

## HUMAN ETHICS APPROVAL DECLARATION

Authors declare human ethics approval was not needed for this study.
